# Iodine and Some of Its Uses in Surgical Work1Original communication published in American Medicine.

**Published:** 1907-06

**Authors:** John Egarton Cannady

**Affiliations:** Surgeon-in-Charge Sheltering Arms Hospital, Hansford, W. Va.


### Iodine and Some of Its Uses in Surgical Work.[1]

By JOHN EGARTON CANNADY, M.D.
Surgeon-in-Charge Sheltering Arms Hospital, Hansford, W. Va.

[*Author's Abstract.*]

IODINE is an exceedingly active substance chemically, and belongs to the halogen group. It possesses great affinities for many substances, and its exact use and sphere of action in the body are unknown. It enters largely into the composition of sea food and animals subsisting on this food contain their share of this evanescent substance. It makes the circuit of the body circulation in a short time and is eliminated in the saliva, urine, and feces.

Senn, in his recent trip among the Esquimaux, noted that iodine is liberally incorporated in the food of these people. He observed the remarkable absence of tumors of all sorts, the exceedingly benign course of spyhilis, the absence of enlarged tonsils, lymphatic glands and goiter. He attributes this immunity to their use of iodised food. Sternberg, Senn, Koch, Schill, Fisher, Behring, Tavel and more recently Kinnaman, have emphasised the value of iodine as an antiseptic. It is certainly the most powerful as well as the least harmful germicide we possess.

Kinnaman has performed an unusually elaborate and careful series of experiments with a view to the determination of the actual antiseptic value of the drug. He made use of a solution containing iodine 2.5 gm., sodium iodid 5.5 gm., sterile water 250 cc., making 1-100 solution. A 1-100 solution of mercuric chlorid acting on a culture of streptococcus pyogenes for 15 minutes, showed a good deal of inhibitory power for the first day but allowed a good growth of streptococci to appear. An exposure of 30 minutes, however, gave no growth. The superiority of iodine is readily evidenced by the fact that a comparatively

1. Original communication published in American Medicine.

weak solution (0.8 per cent.) killed the streptococcus after two minutes exposure. To iodine the staphylococcus is far more resistant than is the streptococcus. While it takes a 1-100 solution five minutes to kill the former, a 1-500 solution is fatal to the latter in two minutes. Dr. Kinnaman's conclusions are that in a solution of iodine varying from 0.2 to 1-6 per cent. we have a germicidal agent of marked potency. Its bactericidal power is far superior to mercuric chlorid, the acknowledged leader of all antiseptics.

The author reports a case of multiple tuberculous abscesses of the muscles of the chest and back, treated by repeated injections of iodoform in olive oil in which the results were most gratifying. He calls attention to the fact that the injection of the emulsion into the joint is naturally followed by a rise of temperature which may last for several days.

The iodoform gauze treatment of puerperal aepsis introduced by the late Dr. Pryor, of New York, is commented on most favorably. The method is considered to be unassailable from a deductive as well as a resultant point of view. Pryor packed the uterus and the retrouterine space with iodoform gauze after thorough curetting and irrigating. The iodoform gauze filling of von Mosetig-Moorhoof has been found to be a most valuable adjunct in the treatment of the circumscribed chronic osteomyelitis.

Aumond and Bonnaire use the following formula for an irrigating solution: iodine 3 gm., potassium iodid 6 gm., water 1,000 gm. They make use of the pure tincture as a local application prior to curetment as a means of partially sterilising the inside of the uterus. Many of the oldtime gynecologists were in the habit of making an application of the plain tincture to the inside of the uterus after curetment.

Iodine in weak solution as an irrigation fluid is of much value in the treatment of suppurative conditions, for example, suppurative arthritis, abscess, empyema, and the like. The author has several times used a one per cent. solution in the treatment of suppurating sinuses and wounds, with the result that there was a prompt disappearance of pus and an abundant formation of healthy granulation tissue. It must not be forgotten that, although iodine is the most harmless of antiseptics, it and its compound iodoform are active agents and as such should be used with caution. They are under circumstances powerfully toxic. It is after injection into serous cavities that the most serious results are seen. The pyogenic membrane lining the tuberculous or pus cavity seems to possess the power of immunity to a marked degree. The old and enfeebled patient will be much more susceptible to the poisonous action than the more robust. It is a well known fact that an individual suffering from septic infection will tolerate much more iodine without the symptoms of poisoning, than one under normal conditions. Rarely there are found persons having so marked an iodiosyncrasy for iodoform that it will act as a poison when exhibited in the usual manner in small amounts.

The author uses a one-half of one per cent. alcoholic solution for purposes of hand disinfection preliminary to operative work, whenever rubber gloves are not worn. The same solution is made use of in the preparation of the site of the operative incision. Rubber gloves are worn as a routine measure in operative work, but in certain number of instances gloves are undesirable; again, in an occasional septic case, a glove may be punctured or torn, and the operator feels the need of some reliable antiseptic for his own sake as well as for the protection of his future patients. The use of this solution simplifies the technic and saves time. The method practised is as follows: first thorough scrubbing with nail brush, green soap and running hot water, going over the hands in a systematic and methodical manner, taking each part in its turn and always following the same order so as to skip no part. Particular attention is paid to the nail folds subungual spaces, and the skin betwen the fingers. Short clipped nails should be cleaned with an orangewood stick, the hands scrubbed again, washing off the soap in running hot water. Remove the residue of the soap with 70 per cent. solution of alcohol, immerse in iodine solution for five minutes, rinse in sterile water. The light-brown stain can be removed by washing in dilute ammonia water after operations, or if left alone will soon disappear.

The results clinically of this method have been superb. In a long series of cases no infection attributable to the hands has occurred.

In his conclusions the author states his belief that the iodine constitutes a near approach to a perfect antiseptic, in that it is nontoxic in effective strength, being one-fourth as poisonous as mercuric chlorid though many times more valuable as a germicide. It does not coagulate albumin or form inert compounds with the tissues. It possesses great penetrating power, is easily prepared, and is stable. A solution of iodine is the most practicable chemical agent we have for the sterilisation of the skin.

---

She (sympathetically)—And that scar on your face is from a bullet wound? How was it that you were shot in the face?

Spanish War Veteran—I foolishly looked back.—Harper's Weekly.

